# Supplementary material for: Beyond locomotion: How specialized motor patterns enable a vertebrate to struggle free from capture
Source: iScience. 2025 Nov 19;28(12):114068. doi: 10.1016/j.isci.2025.114068 (PMC12723385; doi:10.1016/j.isci.2025.114068)
Supplement: Document S1. Figure S1 [file mmc1.pdf]

iScience, Volume *28*

Supplemental information

Beyond locomotion: How specialized motor patterns enable a vertebrate to struggle free from capture

Saeed Farjami, Andrey Palyanov, Hong-Yan Zhang, Valentina Saccomanno, Robert Merrison-Hort, Andrea Ferrario, Roman Borisyuk, Joel Tabak, and Wen-Chang Li

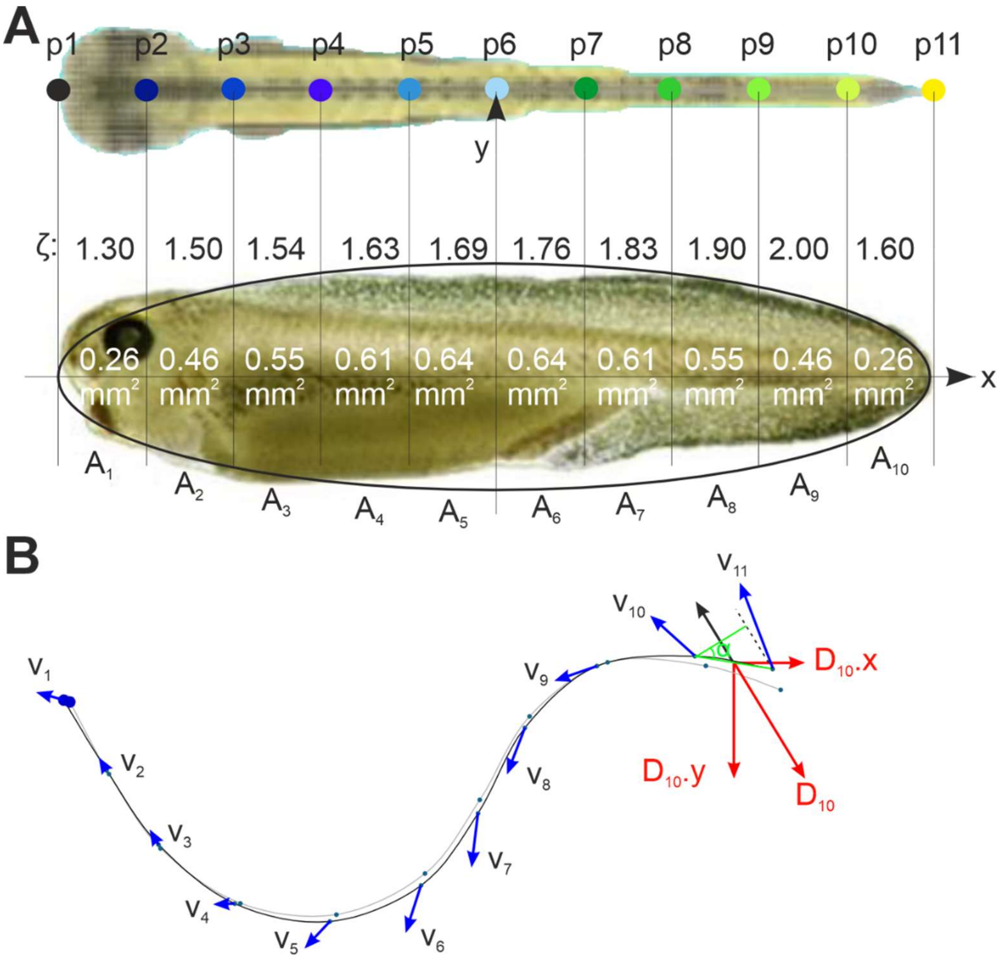

Supplementary Fig.1. Calculating pressure drag generated by VT movement. *Related to STAR Methods.* ***A.*** *Estimating VT surface area. The side view outline of the VT could be approximated as an ellipse with a long axis of 5mm and a short axis of 1.25mm:* $y = \pm 0.63 \times \sqrt{(1 - 0.16x^2)}$*. Half of the area for each of the 10 sections can be calculated by integrating the formula between distances 0, 0.5, 1, 1.5, 2 and 2.5 mm and resultant areas (*$A_{1\text{-}10}$*) for each section are labelled.* ***B****. Diagram showing how to calculate and project drag onto the x and y axes using the section between tracking points p10 and p11, i.e. area* A_{10}*. Thin lines and small dots are tracking positions of two consecutive frames in a video. Assuming the mass centre is at the middle point between p10 and p11, the mass centre velocity (black arrow) is calculated as the average of velocity vectors* v_{10} *and* v_{11}*. Surface area (*A_{10}*) is projected to the plane perpendicular to the averaged velocity vector:* A_{10}**cosine(α). The drag coefficient (ζ) for each section is estimated and given by the shape of its transection. Drag (*D_{10}*) is calculated as:* $D_{10} = \zeta_{10} \times A_{10} \times \cos\alpha \times \rho v^2 \div 2$*, where ρ is the density of the water, v is averaged velocity, ζ is the coefficient of body shape resistance, which depends on the shape and cross-section area of the object against the water flow. Drag is then projected on axes x and y. Total drag on each axis is the summation of drags from all body sections, which are also used to calculate the total drag amplitude* $D_{xyz} = \sqrt{D_x^2 + D_y^2 + D_z^2}$.
